# Confluent and reticulated papillomatosis improvement with tirzepatide-associated weight loss: A case report

**DOI:** 10.1016/j.jdcr.2026.07.054

**Published:** 2026-08-04

**Authors:** Harman Bindra, Gabriella Paquette, Nikki Levin

**Affiliations:** a UMass Chan Medical School, Worcester, Massachusetts; b Department of Dermatology, UMass Chan Medical School, Worcester, Massachusetts

**Keywords:** CARP, confluent and reticulated papillomatosis, GLP-1 receptor agonist, insulin resistance, metabolic dysfunction, obesity, tirzepatide, weight loss

## Introduction

Confluent and reticulated papillomatosis (CARP) of Gougerot and Carteaud is an uncommon dermatosis characterized by hyperpigmented, scaly macules and papules that coalesce into reticulated plaques, most commonly involving the intermammary and interscapular regions of the trunk.^1^ It typically presents in young adulthood, with a slight female predominance. While CARP has been associated with obesity, insulin resistance, and acanthosis nigricans, its exact etiology remains unclear.^2^^,^^3^ Proposed mechanisms include disordered keratinization, bacterial or fungal involvement, and underlying endocrine abnormalities. Although often asymptomatic, the chronic and visible nature of the disease can impact quality of life.

Current treatment strategies include oral tetracyclines, particularly minocycline, topical and oral retinoids, topical vitamin D analogues, and tacrolimus. Despite these options, treatment outcomes remain variable, and long-term disease control can be challenging.

The association of CARP with obesity and insulin resistance suggests a possible role for metabolic dysregulation in disease pathogenesis.^4^^,^^5^ A single case report demonstrated resolution of CARP following a large weight loss after bariatric surgery in a patient who was refractory to both antifungal and antibacterial therapies.^6^ Here, we present a case of clinical resolution of CARP in the setting of weight loss associated with GLP-1 receptor agonist therapy, and recurrence following discontinuation, highlighting a potential novel therapeutic approach.

## Case report

A 29-year-old female patient with no significant dermatologic history presented to the dermatology clinic in March 2022 while pregnant, reporting a several-year history of rash involving the chest that worsened during pregnancy (Fig 1). Physical examination demonstrated reticulated brown plaques on the medial chest consistent with CARP.

**Fig 1:**
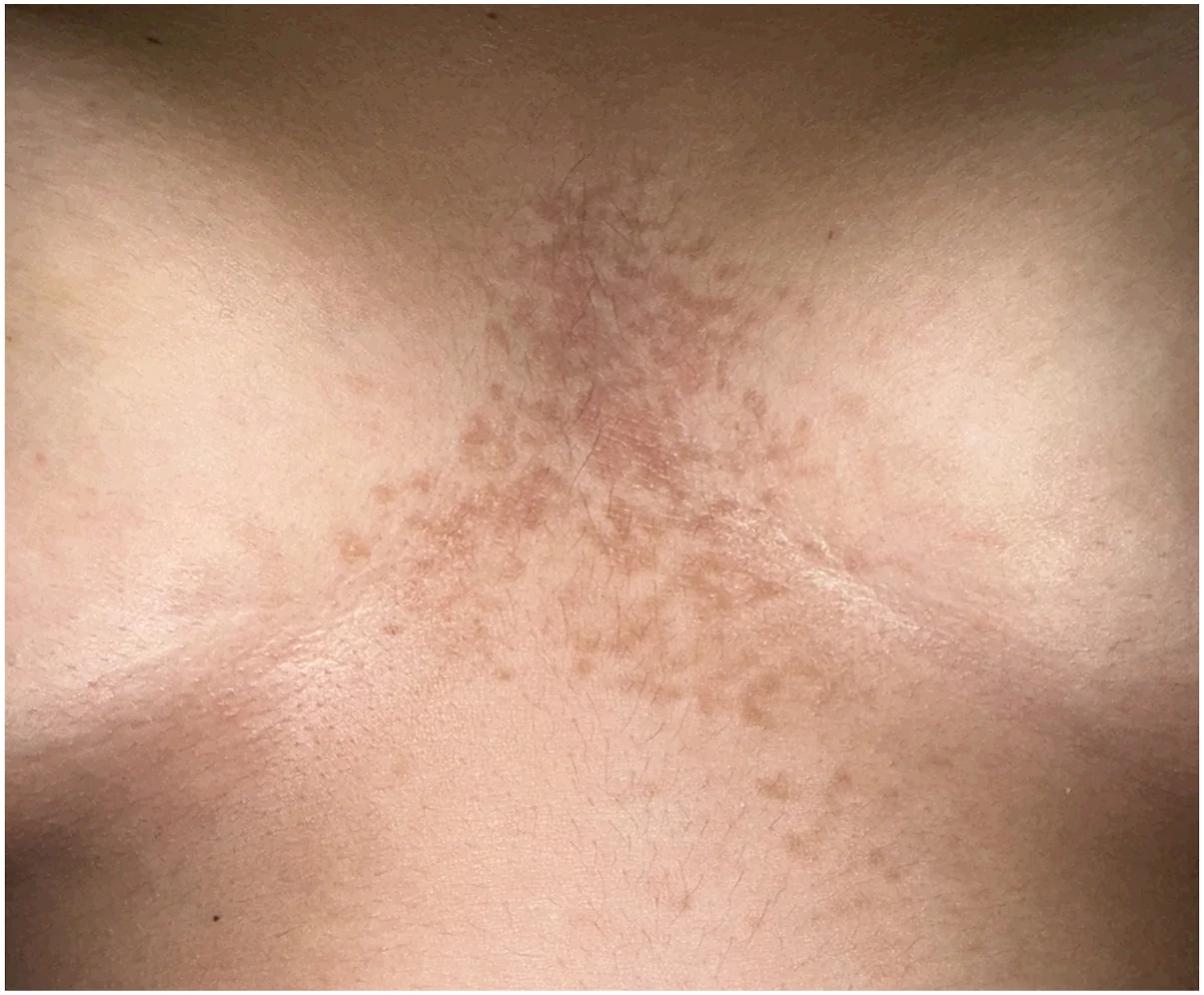
Hyperpigmented reticulated papules and plaques on the central chest consistent with Confluent and reticulated papillomatosis of Gougerot and Carteaud (CARP) at time of patient’s initial presentation September 2024. *CARP*, Confluent and reticulated papillomatosis.

From 2022 through 2025, the patient underwent multiple treatment trials with limited or transient improvement, including hydrocortisone 2.5% cream, ketoconazole cream, topical tacrolimus, topical metronidazole, topical econazole, and oral doxycycline. There were intermittent disruptions in therapy related to pregnancy and breastfeeding. Despite partial responses, symptoms consistently recurred following treatment discontinuation.

During this period, the patient also pursued medical weight management and was started on tirzepatide 5 mg weekly in July 2025, increasing to 7.5 mg weekly after 1 month. About 1 month later (August 2025), after losing between 2 and 5 kg, the patient noted significant improvement in her CARP (Fig 2). She maintained improvement in her CARP and lost a total of 18 kg by the time tirzepatide was tapered and discontinued 3 months later (November 2025) after a total of 5 months of therapy, due to hair loss. Her CARP remained clear until December 2025, 2 to 3 weeks after discontinuation, in the setting of 2–5 kg of weight gain. At telephone follow-up in April 2026, the patient reported a gain of 9 kg since GLP1 agonist discontinuation, with partial recurrence of the rash and mild pruritus (Fig 3).

**Fig 2:**
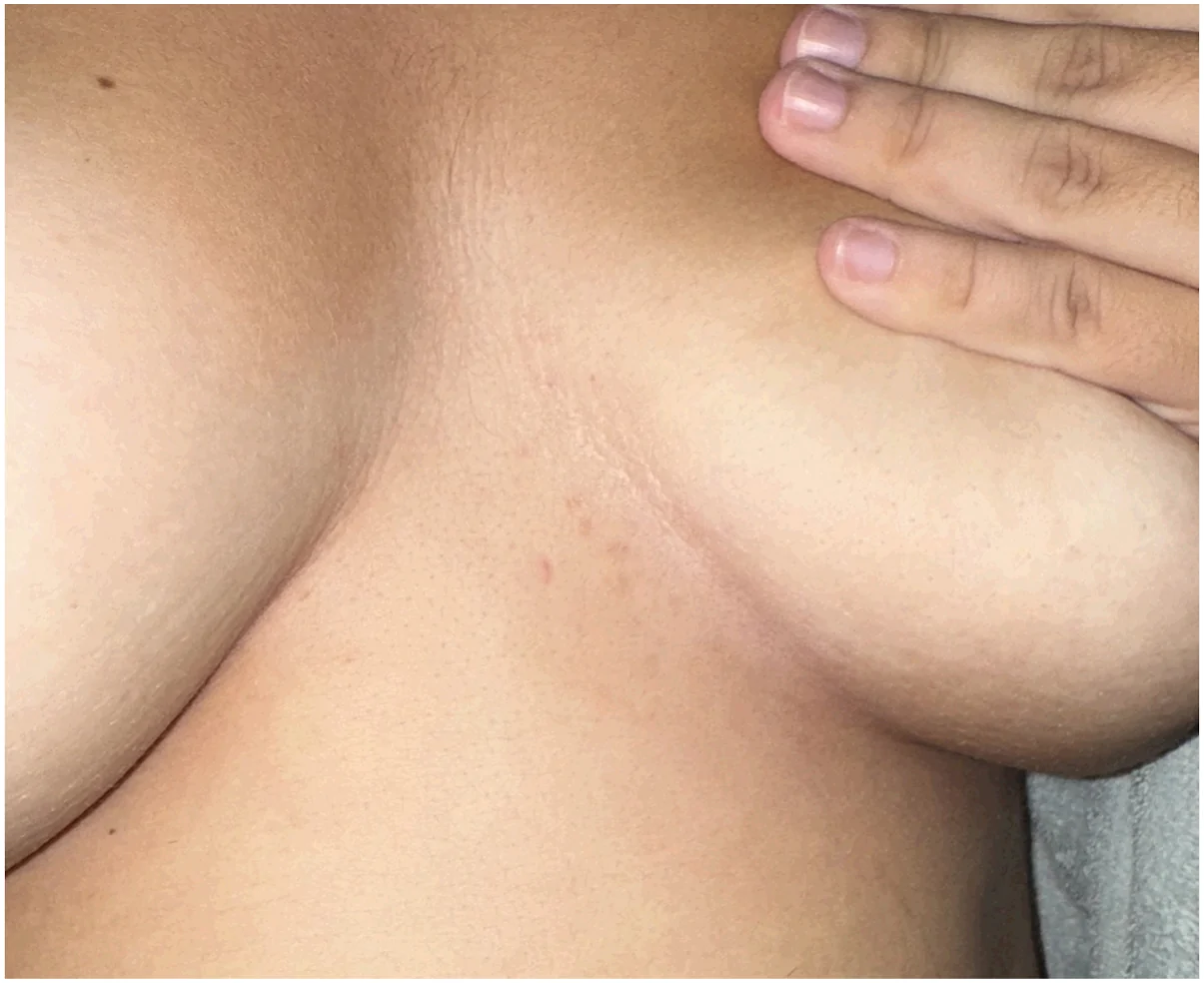
Near complete clearance CARP at follow-up August 2025 1 month after starting tirzepatide. CARP, Confluent and reticulated papillomatosis.

**Fig 3:**
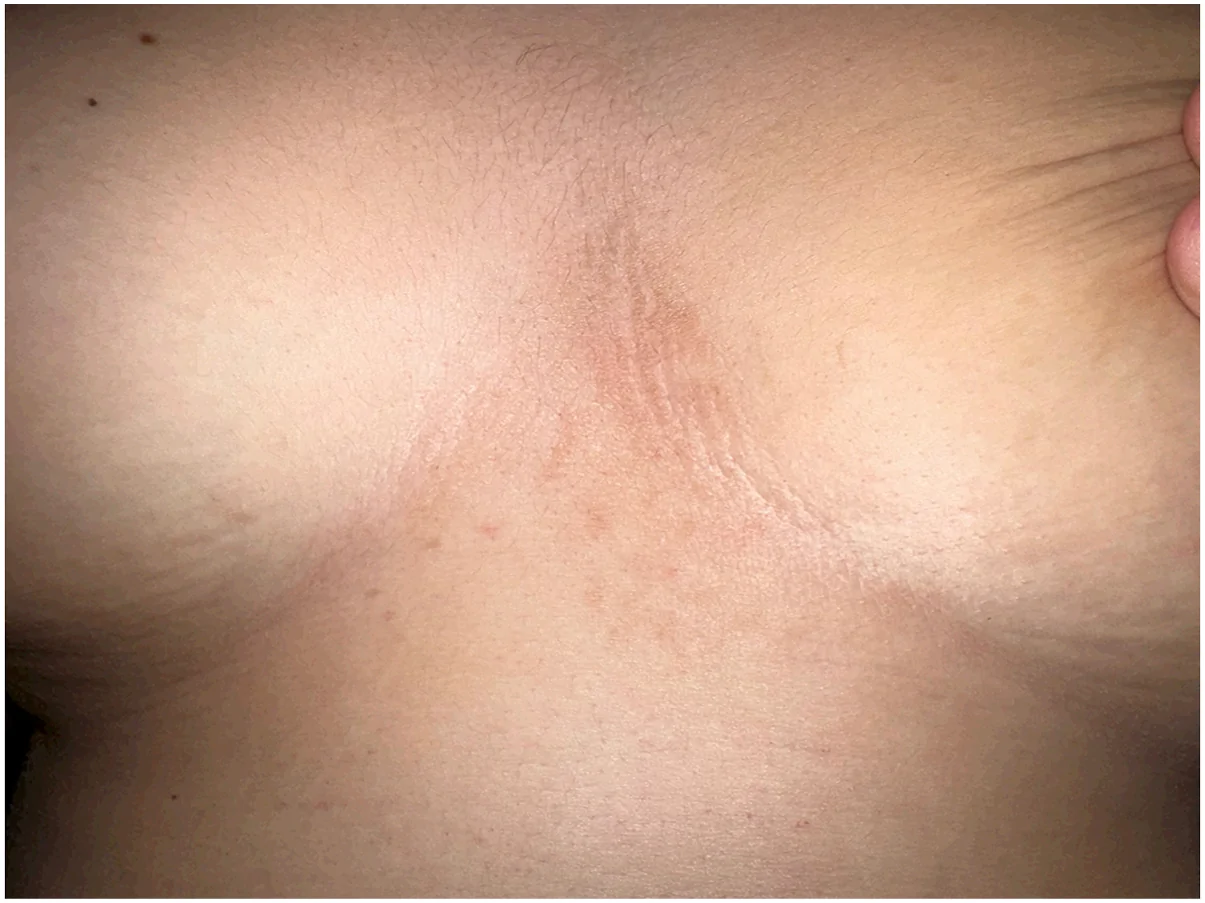
Recurrence of CARP in June 2026 4 months after discontinuation of tirzepatide associated with an approximately 9 kg weight gain. *CARP*, Confluent and reticulated papillomatosis.

## Discussion

This case highlights a potential association between metabolic dysfunction and CARP. Although the pathogenesis of CARP remains incompletely understood, proposed mechanisms include insulin resistance and hyperinsulinemia, which may promote epidermal proliferation by activating insulin-like growth factor pathways. Obesity and acanthosis nigricans, both conditions associated with insulin resistance, have also been reported in patients with CARP.

GLP-1 receptor agonists could potentially improve CARP through both weight reduction and improved insulin sensitivity, reducing the proliferative stimulus contributing to keratinocyte dysfunction. Notably, this patient experienced improvement in her rash within months of initiating therapy, and before substantial weight loss, suggesting that the therapeutic effect of GLP-1 receptor agonists may not be mediated solely through weight reduction. In this patient, the temporal association between GLP-1 therapy, early disease improvement, significant weight loss, medication discontinuation, and subsequent symptom recurrence further supports a possible metabolic contribution to disease activity. Given the limited literature on metabolically targeted therapies in CARP, further investigation is needed to better define the role of GLP-1 receptor agonists and other insulin-sensitizing agents in disease management.

## Conflicts of interest

None disclosed.
